# Ready for the First Cardiac Arrest: A Teaching Intervention Using a Video-Based Simulation to Increase Confidence and Preparedness to Perform Cardiac Arrest Roles in Newly Qualified Doctors

**DOI:** 10.7759/cureus.96612

**Published:** 2025-11-11

**Authors:** Leora Y Marcus, David Mafullul, Harine Baalamurugan, Samson Arokiyanathan

**Affiliations:** 1 West Herts Initiative in Simulation Education and Research (WiSER), Watford General Hospital, Watford, GBR; 2 Medicine, Watford General Hospital, Watford, GBR; 3 Medicine, West Hertfordshire Teaching Hospitals NHS Trust, Watford, GBR; 4 Orthopaedics, West Hertfordshire Teaching Hospitals NHS Trust, Watford, GBR

**Keywords:** clinical simulation, doctor confidence, newly qualified doctor, preparation for cardiac arrest, teaching pilot, video simulation

## Abstract

Background

Many newly-qualified doctors take time to grasp the knowledge and skills needed for managing a cardiac arrest. One intervention which can reduce the time taken to feel confident is video-based simulation training that covers essential information, including role allocations and local Trust-specific procedures, which would only otherwise be learned ad hoc. A video simulation course offers a quick, efficient teaching format which can be easily incorporated into induction week. It serves to help doctors feel more confident and prepared for managing cardiac arrests from the very start of their careers. This study aimed to use video-based simulation training at Watford General Hospital (UK) to, first, assess newly qualified doctors’ knowledge of cardiac arrest roles by measuring changes in role identification accuracy, and, second, to improve newly qualified doctors’ confidence and preparedness for a cardiac arrest at the start of their careers, measured using self-reported confidence scores.

Methodology

In total, 70 doctors participated in this teaching intervention. Of these, 46 (65.7%) completed an anonymous pre-session questionnaire, and 37 (52.9%) completed a post-session questionnaire. The questionnaires assessed the participants’ knowledge, preparedness, and confidence to participate in roles at cardiac arrest. The teaching intervention consisted of a video-based simulation and a debrief facilitated by senior resident doctors. It was supported by a presentation focusing on job roles and Trust-based procedures. Knowledge of cardiac arrest roles was measured as the number of distinct roles participants could correctly identify. Confidence and preparedness to participate in these roles were self-reported on a 10-point Likert scale. Responses were analysed using the Mann-Whitney U test for Likert scales and using descriptive statistics for role identification.

Results

Overall, 35 (97.2%) respondents in the post-session questionnaire felt the session increased their confidence in managing a cardiac arrest. Identification increased for all roles, except for compressions and defibrillation. Their identification of key roles such as timekeeper, scribe/documenting, and runner/bringing equipment improved by 42.6%, 23.1%, and 30.8%, respectively. These roles had been among the least identified in the pre-session questionnaire. Post-session, doctors felt they were better prepared to attend a cardiac arrest (n_1_ = 46, n_2_ = 36, U = 323.5, p < 0.00001), and that they could contribute more effectively (n_1_ = 45, n_2_ = 37, U = 296.5, p < 0.00001).

Conclusions

The pilot session demonstrated that video-based simulation with a debrief was an effective method to improve newly qualified doctors’ knowledge and confidence in participating in the roles within a cardiac arrest team. Participants described the pilot as a valuable intervention in supporting them in their first cardiac arrests. Further research is needed to evaluate if these results will translate into improved performance in cardiac arrest outcomes.

## Introduction

The transition from medical student to doctor is a daunting and often stressful experience, marked by a sudden increase in responsibility and skills. Studies show graduates feel unprepared for this change. Much of newly qualified doctors’ early learning occurs ‘on the job’ which includes ward work, learning the physical and organisational structure of the hospital, and the management of the acutely unwell patient, all of which differ between NHS Trusts [1,2]. The gaps in this practical knowledge lead to the annual ‘Black Wednesday’, when hospital mortality increases in the first week of August, when newly qualified doctors start work [3]. The increased mortality rate reflects reduced experience of newly incoming doctors and other staff members in delivering hospital care, and likely includes managing a cardiac arrest.

A cardiac arrest call demands effective teamwork and a sound understanding of the Advanced Life Support (ALS) algorithm and Trust processes to optimise outcome. This high-pressure situation exposes participants’ uncertainties regarding equipment location, the roles involved, and the medical grades which are expected to occupy each role. Previous surveys suggest 73% of trainee doctors find cardiopulmonary resuscitation (CPR) stressful, and the lack of role allocation is a common cause of stress [4]. It is, therefore, unsurprising that a newly qualified doctor finds attending a cardiac arrest a stressful experience, especially at the start of their career. In most Trusts, a newly qualified doctor could be attending a cardiac arrest call from their first day, yet a qualitative analysis suggests experience from working for three to six months is needed to acquire crucial skills and feel more confident to manage an acutely unwell patient [5].

A method of teaching to reduce this three-to-six-month time lag is simulation training. In this, participants can practice and develop clinical competency by experiencing, in a safe environment, an imitation of emergency situations. Structured debriefing allows reflection, feedback, and improvement in the participants’ performance. Several systematic reviews show that high-fidelity simulation (HFS), when compared with traditional methods, is associated with greater improvement in learner satisfaction, knowledge, and skill performance [6,7]. However, access to HFS training requires significant resources, including specialist equipment, facilitators, and a half-to-a-full-day schedule that only teaches a small group, all of which may be difficult to deliver during a busy induction week’s schedule. Video-based low-fidelity simulation (v-LFS) is quicker and scalable to a larger cohort, providing a practical alternative. Evidence shows v-LFS can improve knowledge in the context of medical emergencies, although the magnitude of this improvement and longer-term retention of this knowledge remains better for HFS [8,9]. However, v-LFS sessions can easily be held during doctors’ mandatory induction week, reaching all incoming doctors, and therefore providing essential training in managing emergencies, including cardiac arrests.

Aims

This teaching pilot aims to assess and improve identification accuracy and knowledge of cardiac arrest roles among newly qualified doctors at Watford General Hospital, UK (WGH). This pilot aims to improve the doctors’ confidence and preparedness, measured in self-assessed confidence scores, to participate in roles in a cardiac arrest from the start of their employment. Our pilot has been run in conjunction with the West Herts Initiative in Simulation Education and Research Team (WiSER) and the Foundation Team (this team manages newly qualified doctors).

## Materials and methods

Participants

This pilot was taught to newly qualified doctors at WGH during their induction week. The cohort of doctors had completed ALS training a week before the session and had not started ward work.

Ethical considerations

This study was considered low-risk and did not require formal ethical approval. All participants were informed that the results would be used for publication and that their participation was voluntary. No data were collected from individuals who chose not to participate. This research project involved healthcare staff by virtue of their professional role and presented no material ethical issues. It did not require a Research Ethics Committee review as per the Governance Arrangements for Research Ethics Committee.

Study design

Participants completed an anonymous pre-session questionnaire, which assessed their knowledge. This questionnaire included short-answer questions and a 10-point Likert scale to quantify their preparedness and confidence in participating in specific cardiac arrest roles. Participants watched a five-minute video simulation of a cardiac arrest, which showed poor performance in coordination, communication, and inadequate allocation of job roles. This was followed by a 20-minute debrief led by two senior resident doctors. The debrief was aided by a presentation which discussed job roles needed during a cardiac arrest. Focus was given to roles commonly performed by newly qualified doctors, such as scribing, requesting investigations, prescribing, compressions, running blood gas, timekeeping, and vascular access. After this, the cohort watched a two-minute video of a simulated cardiac arrest, demonstrating good allocation of job roles and better overall performance. The cohort then participated in another debrief highlighting why the second video had a better performance outcome. Finally, the cohort completed a post-session questionnaire with similar questions to the pre-session questionnaire. Answers to these questionnaires were compared.

Materials

The session was run in a lecture theatre. Anonymous responses to the questionnaires were recorded via Google Forms (Appendices). The two cardiac arrest videos were recorded by the WiSER team. The first video had poor role allocation and did not adhere to the Resuscitation Council UK guidelines on the ALS Algorithm. The second video followed the ALS Algorithm and had clear role allocation, which led to a quicker identification of cardiac arrest to CPR and defibrillation time compared with the first video. The presentation consisted of a slide show, where each slide was dedicated to a discrete role and included a brief summary of vital information. This was used to aid the debrief. Facilitators followed the three-phase debrief model.

Data coding and scoring

Identification of job roles was assessed as the number of distinct job roles described in a short-answer response. Examples of answers accepted and not accepted are summarised in Table 1.

**Table 1:** Examples of job roles accepted and not accepted in response to the question ‘what roles are needed in a cardiac arrest call?’

Question	Acceptable answers (1 point for each)	Examples of answers not accepted
What roles are needed in a cardiac arrest call?	ABCDE Assessment (examination of the patient)	Oxygen
	Access/Taking bloods	CPR
	Airway/Ventilation	Anaesthetist/Doctor/Nurse
	Compressions	
	Defibrillation/Automated external defibrillator (AED)	
	Documentation/Scribe/Gathering information	
	Ordering/Administering medication	
	Requesting investigations	
	Runner/Running the blood gas/Calling a cardiac arrest/Bringing a crash trolley	
	Timekeeping	
	Vital signs/Observations	

Data analysis

Population data, in the form of a Likert scale, was statistically analysed using a Mann-Whitney U test as the data was ordinal. As questionnaire responses were anonymous, data were analysed unpaired. Open-ended responses were manually reviewed, with each acceptable key term coded as correct, creating a binary dataset for quantitative analysis. Blank responses to short-answer questions were included in the analysis under the assumption that a blank answer meant the participant did not know the answer.

## Results

Participant demographics

In total, 70 doctors attended this teaching session, of whom 46 (65.7%) completed the pre-session questionnaire and 37 (52.9%) completed the post-session questionnaire. All doctors were due to start working on the wards within three days.

Knowledge of cardiac arrest roles

Participants listed their answers for this open-ended question (Table 2). Among the most poorly identified roles pre-session were timekeeping, vascular access/bloods, and documentation (8.7%, 26.1% and 28.3%, respectively). Post-session, identification increased for all roles except compressions and defibrillation. Timekeeping was associated with the greatest increase in identification from 8.7% to 51.4%. Post-session, the identification of the role of the runner increased by 30.8%, and the role of documenting by 23.1%. The average number of correct answers per participant increased from 3.1 pre-session to 4.0 post-session.

**Table 2:** Number of doctors who identified each cardiac arrest role in the pre- and post-session questionnaires. The percentage of respondents who mentioned each role is shown in parentheses.

Roles	Pre-session (n = 46)	Post-session (n = 37)
Compressions	19 (41.3%)	10 (27.0%)
Timekeeping	4 (8.7%)	19 (51.4%)
Defibrillation	23 (50.0%)	13 (35.1%)
Access/Bloods	12 (26.1%)	13 (35.1%)
Documentation/Scribe	13 (28.3%)	19 (51.4%)
Leader	27 (58.7%)	22 (59.5%)
Runner/Bringing equipment	2 (4.3%)	13 (35.1%)
Calling a cardiac arrest/for help	2 (4.3%)	3 (8.1%)
Airway	15 (32.6%)	16 (43.2%)

Feelings of preparedness and the ability to contribute effectively during cardiac arrest calls

**Figure 1:**
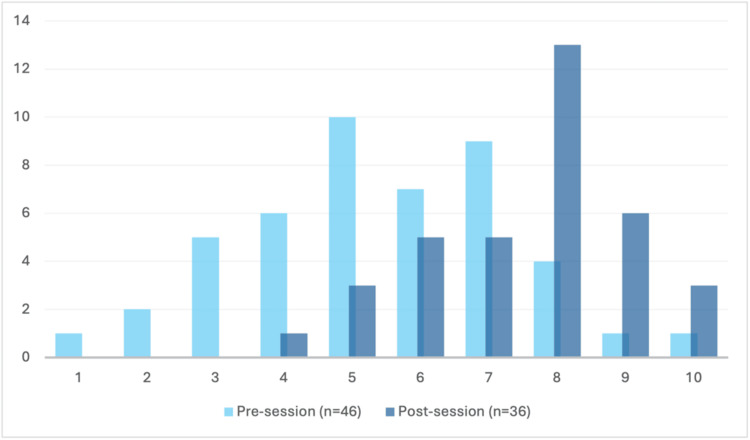
Responses to the question ‘I feel I am well prepared to attend a cardiac arrest call’. Feeling of preparedness to attend a cardiac arrest call among participating doctors before (light blue) and after (dark blue) the session. Doctors anonymously ranked their feelings on a 10-point Likert scale from 1 (‘not at all prepared’) to 10 (‘very prepared’), as represented along the x-axis. The number of individuals is represented along the y-axis. Doctors felt significantly more prepared post-session (n_1_ = 46, n_2_ = 36, U = 323.5, p < 0.00001).

**Figure 2:**
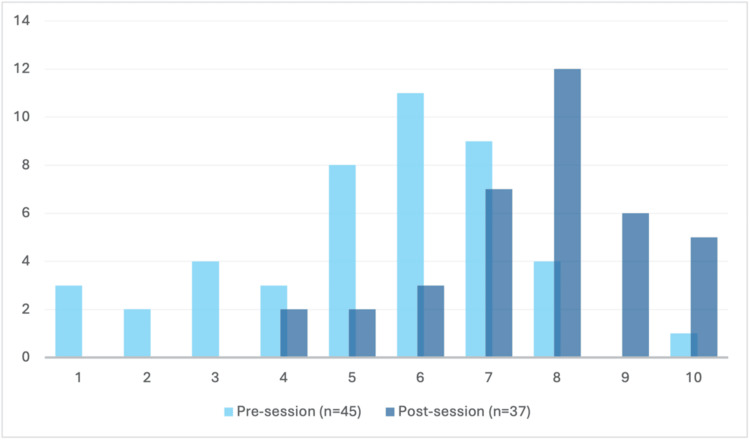
Responses to the question ‘I feel I can contribute effectively in an arrest call’. The extent to which participating doctors felt they could contribute effectively at a cardiac arrest call before (light blue) and after (dark blue) the session. Doctors anonymously ranked their feelings on a 10-point Likert scale from 1 (‘not at all confident’) to 10 (‘very confident’), as represented along the x-axis. The number of individuals is represented along the y-axis. Doctors felt significantly more confident post-session (n_1_ = 45, n_2_ = 37, U = 323.5, p < 0.00001).

Participants felt more prepared to attend a cardiac arrest call (n_1_ = 46, n_2_ = 36, U = 323.5, p < 0.00001) and thought that they could contribute more effectively at a cardiac arrest call (n_1_ = 45, n_2_ = 37, U = 296.5, p < 0.00001) after the session, as seen in Figure 1 and Figure 2. Modal results increased from 5/10 to 8/10 for preparedness to attend a cardiac arrest and from 6/10 to 8/10 for confidence to contribute to an arrest call. Overall, 97.2% respondents in the post-session questionnaire answered that the session increased their confidence in managing the unwell patient.

Confidence in documenting

**Figure 3:**
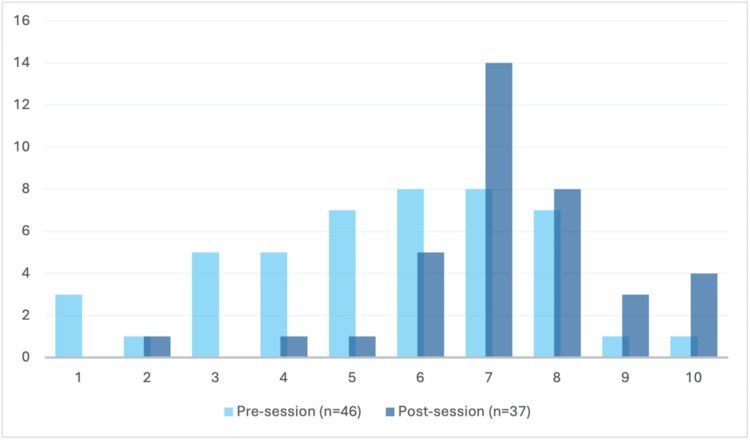
Responses to the question ‘I feel confident in documenting’. The feeling of confidence in documenting a cardiac arrest among participating doctors before (light blue) and after (dark blue) the session. Doctors anonymously ranked their feelings on a 10-point Likert scale from 1 (‘not at all confident’) to 10 (‘very confident’), as represented along the x-axis. The number of individuals is represented along the y-axis. Doctors felt significantly more confident post-session (n_1_ = 46, n_2_ = 37, U = 443, p = 0.0002).

After participating in this teaching pilot, doctors felt more confident in documenting (n_1_ = 46, n_2_ = 37, U = 443, p < 0.0002). Figure 3 demonstrates a positive shift of responses, with only two (8%) responses scoring less than 5 after the teaching session, compared to 14 (45%) before the session. Pre-session, self-reported confidence was more widely distributed, with 21 (45.7%) participants rating between 1 and 5, and 25 (54.3%) participants rating between 6 and 10 (n₁ = 46). Post-session, three (8.15%) participants rated their confidence between 1 and 5, with the remaining 34 (91.9%) participants rating between 6 and 10 (n₂ = 37). Mean response increased from 5.5 to 7.3.

Confidence in performing chest compression

**Figure 4:**
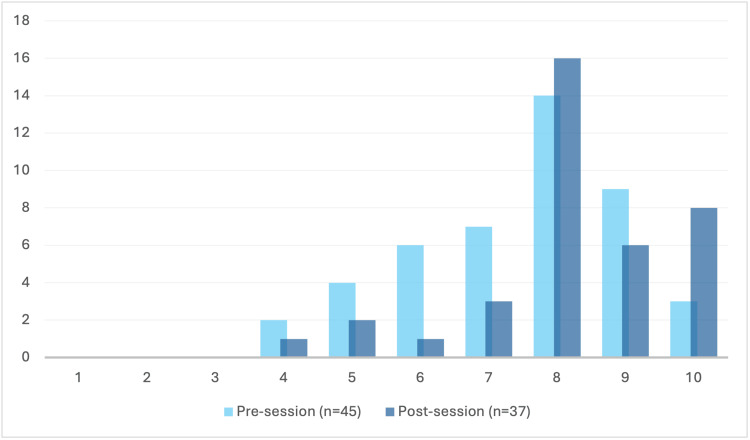
Responses to the question ‘I feel confident in doing chest compressions’. The feeling of confidence in performing chest compressions among participating doctors before (a) and after (b) the session. Doctors anonymously ranked their feelings on a 10-point Likert scale from 0 (‘not at all confident’) to 10 (‘very confident’), as represented along the x-axis. The number of individuals is represented along the y-axis. Doctors felt significantly more confident post-session (n₁ = 45, n₂ = 37, U = 611, p < 0.005).

Following the teaching pilot, participants reported increased confidence in performing chest compressions (n₁ = 45, n₂ = 37, U = 611, p < 0.05), as shown in Figure 4. Although the modal response remained at 8/10, more participants scored 8 or above post-session.

Identification of the correct locations of the blood gas machine at WGH

This was an open-ended question where respondents listed locations of blood gas machines in WGH. In the pre-session questionnaire, five (10.8%) left the answer blank, five (10.8%) responded ‘did not know’, and the remaining 36 (78.3%) included at least one correct answer (n_1_ = 46). Post-session, all respondents gave at least one correct answer, and six left the answer blank (n_2_ = 37).

Qualitative feedback

The final question in the post-session questionnaire was optional feedback for participants to comment on anything they had learned. Overall, nine out of sixteen responses mentioned learning about job roles as something achieved from the teaching session, with many positive comments regarding how the session aided them, specifically in documenting. In fact, feedback overall was very positive. Participants commented that the session was ‘very useful and helpful’ and the ‘best class we’ve had at induction’.

## Discussion

Confidence and preparedness to participate in a cardiac arrest and manage the unwell patient

This teaching pilot helped newly qualified doctors feel significantly more prepared to attend a cardiac arrest call (n_1_ = 46, n_2_ = 36, U = 323.5, p < 0.00001), and significantly more prepared to contribute more effectively during the event (n_1_ = 45, n_2_ = 37, U = 296.5, p < 0.00001). Before the teaching, self-reported preparedness and confidence were widely dispersed across the Likert scale (Figures 1, 2). Following the intervention, this distribution shifted positively, where zero participants scored below 4 in confidence or preparedness. These findings suggest that incoming doctors’ preparedness for cardiac arrest was a pre-existing weakness, consistent with previous reviews. While several narrative studies evaluated factors influencing new graduate doctors’ preparedness for practice [10,11], few evaluated preparedness or confidence, specifically in the context of a cardiac arrest, and even fewer quantified the efficacy of a specific intervention (e.g. a teaching programme) to make improvements [12,13]. Our teaching pilot showed significant improvement in the participants’ self-confidence, with almost all reporting that the session improved their confidence in managing the unwell patient.

Identification of job roles in a cardiac arrest

**Figure 5:**
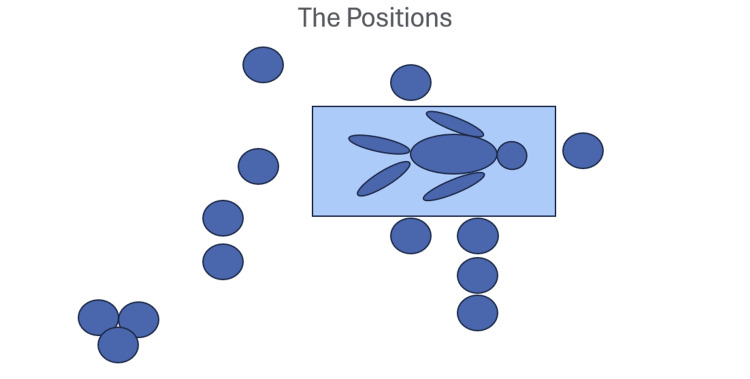
Diagram used in the teaching session to prompt participants about the job roles in a cardiac arrest and where to stand.

Following this teaching intervention, newly qualified doctors recognised a greater number of cardiac arrest roles. Minimal, if any, research focuses on doctors’ ability to identify the discrete roles in cardiac arrests. Most research discusses only the importance of role allocation and how this leads to a better outcome and a less stressful experience for doctors [4,14]. With this in mind, our pilot concentrates on how each of the roles is carried out. Figure 5 is an example of a visual aid used in the debrief to prompt discussion about the roles. Roles for which identification increased the most included timekeeping, scribing, and runner. This increase reflects the session’s focus on these roles, which are expected of newly qualified doctors in our Trust. As they are relatively uncomplex, low-risk tasks, compared to other roles (such as defibrillation or team leader), scribing, documenting, and runner are completed by newly qualified doctors frequently. These roles play a critical part in optimising efficiency and outcome in a cardiac arrest. For example, timekeeping was identified by 42.7% more after the debrief. Awareness of this role is important, as a study showed that implementing two timekeeper roles for a two-minute rhythm check and adrenaline administration is associated with reduced deviation from ALS guidelines and a higher return of spontaneous circulation rate [15]. The runner role has several benefits. The runner can promptly run a blood gas and therefore identify reversible causes of an arrest sooner and liaise with family members and other teams. Identification of the runner as a role increased by 31% post-session. During the debrief, we took advantage of a discussion around the role of runner to highlight how to call an in-hospital emergency, and where within WGH a blood gas can be run. The post-session increase in correct responses demonstrates that participants had effectively learned this critical operational information, suggesting that targeted teaching can improve awareness of essential logistics and thus lead to increased preparedness.

Confidence in documenting

The findings from this teaching pilot demonstrate a statistically significant increase in newly qualified doctors’ confidence in documentation. Figure 3 shows a markedly positive shift in respondents’ confidence in documenting, which is consistent with the statistical analysis (n_1_ = 46, n_2_ = 37, U = 443, p = 0.0002). This suggests newly qualified doctors need support with scribing. Medical school did not prepare them for this, despite it being a common role for newly qualified doctors. Mardegan et al. confirm this, because in their findings, only 22.7% of their junior doctor cohort felt confident to document, and following a teaching session, this increased to 89.5% [14]. Before our teaching pilot, there was a mean response of 5.5 in confidence to document. An extensive discussion was held on scribing and its essential features, such as the timings of the administration of drugs and when shocks were given. The results increased to 7.3, demonstrating the intervention had a meaningful impact on the participants. Accurate documenting of a cardiac arrest is important both medicolegally and in maintaining continuity during post-resuscitative care [14]. By making the participants more confident in documentation, perhaps they will be more likely to volunteer to document at the first cardiac arrest they attend and be a valuable contributing member to the team.

Confidence in performing chest compressions

An unexpected result from this teaching pilot was that newly qualified doctors felt significantly more confident in performing chest compressions (n_1_ = 45, n_2_ = 37, U = 611, p < 0.05). Pre-session, our results varied more for preparedness to complete chest compressions in comparison to previous studies. For example, Menzes et al. found 89% and Bin Sukor et al found 97.4% of their cohort were confident in chest compressions at the start of their research [4,16]. However, both cohorts in this study ranged across medical grades and experience at cardiac arrest, unlike our cohort, who have yet to work in a cardiac arrest team. Although our session did not cover technical aspects of chest compressions (e.g. depth, rate, recoil, and location), the importance of the following was covered: initiating compressions as soon as possible after recognising cardiac arrest, minimising interruptions, and regularly swapping compressors. These teaching points were likely to have contributed to the observed increase in confidence in performing chest compressions.

This teaching pilot was successful in increasing confidence and recognition of cardiac arrest roles, but it only covered basic points. However, it is important to note that confidence does not necessarily equate to competence. In future, to evaluate if this session improves competency, direct observation of performance during HFS could be performed to determine true skill acquisition, and this could be compared between cohorts who did and did not participate in a prior v-LFS session. Nevertheless, several studies demonstrate that v-LFS methods help improve knowledge in the context of managing medical emergencies [8,9].

Limitations

The results were obtained from a single small sample, so they may not represent incoming doctors across the whole cohort or nationwide. The assessment relied on self-reported answers. There is, first, a response bias, as fewer participants completed the post-session questionnaire. Another example of the limitation of the self-reported answers is that the identification of compressor and defibrillation roles decreased in the post-session questionnaire. This result may be explained by doctors focusing on listing roles for which they learned the most during the session. Conversely, compressions and defibrillation are arguably the most obvious roles within a cardiac arrest team, and participants may have assumed this did not warrant listing. This finding highlights the limited use of open-ended questions to assess knowledge, especially when the same participants are completing the same questionnaire at the start and end. This could underestimate their knowledge. Another underestimation example is blank answers, which were assumed to indicate that the participant did not know. However, there could be alternative reasons, such as accidentally skipping the question or reluctance to type lengthy responses. The Likert scale question had a much higher uptake of responses. To mitigate this in the future, the questionnaire could be adjusted so participants can only move on from an open-ended question once an answer is filled in, or to make it a short-answer question to ensure more accurate data capture. It is uncertain if an increase in confidence and preparedness translates to improved competence and performance. This is because there is only basic knowledge testing, not covering in detail what is involved in each role. In addition, this study does not involve direct observation of participants completing a discrete job role. Therefore, our study is limited in interpreting whether they fully acquired new skills in managing cardiac arrest. In future studies, HFS can be used to assess skill acquisition along with confidence and preparedness.

## Conclusions

This pilot session aimed to support newly qualified doctors for their first months on the wards when attending a cardiac arrest. To support them, our teaching session covered the role allocations, specifically emphasising roles which, as newly qualified doctors, they could perform to ensure they are a valued member of the team. Our session highlighted that the participants felt unprepared and unconfident to contribute effectively to a cardiac arrest before the teaching pilot. The post-session questionnaire showed the participants had significant improvement both in the identification of job roles and in their own self-view of preparedness and confidence. Further work is needed to evaluate if increased preparedness and confidence translate to improved performance outcomes in cardiac arrest.

## Figures and Tables

**Figure 6:**
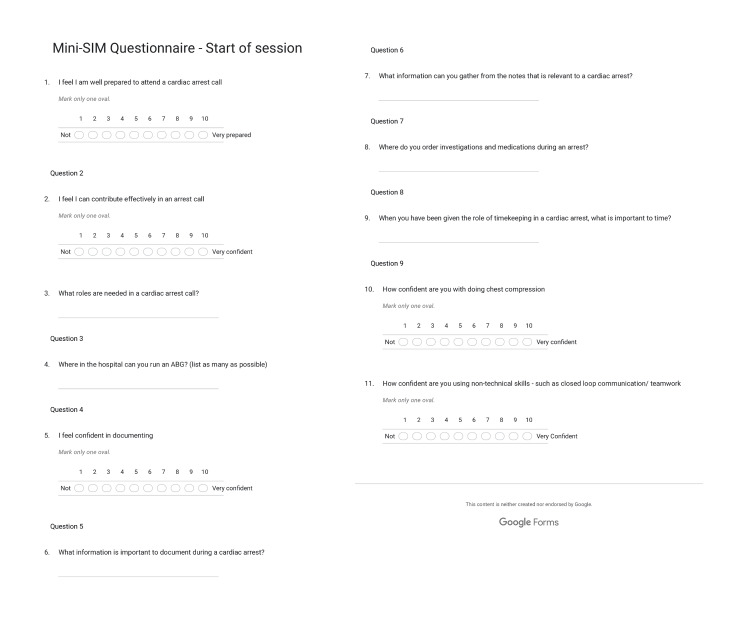
Pre-session questionnaire.

**Figure 7:**
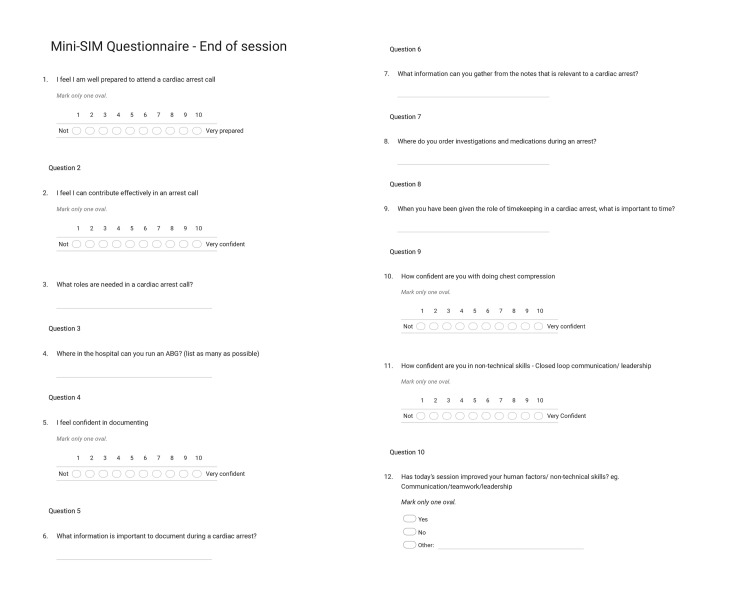
Post-session questionnaire.
